# Regulation of gene expression in rats with spinal cord injury based on microarray data

**DOI:** 10.3892/mmr.2015.3670

**Published:** 2015-04-23

**Authors:** GUOQIANG CHEN, XIUTONG FANG, MENG YU

**Affiliations:** Department of Orthopedic Surgery, Beijing Shijitan Hospital Affiliated to Capital Medical University, Beijing 100038, P.R. China

**Keywords:** spinal cord injury, differentially expressed genes, protein-protein interaction network, transcription factors, microRNAs

## Abstract

The present study aimed to investigate the molecular mechanisms of spinal cord injury (SCI) in rats. First, the differentially expressed genes (DGEs) were screened based on GSE45006 microarray data downloaded from Gene Expression Omnibus using the significant analysis of microarray (SAM) method. Screening was performed for DEGs which were negatively or possibly correlated with time and subsequently subjected to gene ontology (GO) functional annotation. Furthermore, pathway enrichment analysis using the Kyoto Encyclopedia of Genes and Genomes was also performed. In addition, a protein-protein interaction (PPI) network was constructed using the Search Tool for the Retrieval of Interacting Genes/Proteins database. Finally, GeneCodis was used to seek transcription factors and microRNAs that are involved in the regulation of DEGs. A total of 806 DEGs were upregulated and 549 DEGs were downregulated in the rats with SCI. Cholesterol metabolism-associated genes (e.g. *HMGCS1*, *FDFT1* and *IDI1*) were negatively correlated with time, while injury genes (e.g. *SERPING1*, *C1S* and *RAB27A*) were positively correlated with time after SCI. PCNA, MCM2, JUN and SNAP25 were the hub proteins of the PPI network. The transcription factors LEF1 and SP1 were observed to be associated with the regulation of two DEGs that were involved in the cholesterol-associated metabolism as well as injury responses. A number of microRNAs (e.g. miR210, miR-487b and miR-16) were observed to target cholesterol metabolism-associated DGEs. The hub genes *PCNA*, *MCM2*, *JUN* and *SNAP25* presumably have critical roles in rats with SCI, and the transcription factors LEF1 and SP1 may be important for the regulation of cholesterol metabolism and injury responses following SCI.

## Introduction

Spinal cord injury (SCI) refers to any injury to the spinal cord, and the symptoms may vary widely, from pain to paralysis to incontinence. The more serious and profound consequences of SCI are microscopic events following initial tissue injury, including inflammation, necrosis, apoptosis and glial scar formation (1). Microarrays have been used to unveil the short-and long-term responses to SCI at the molecular level, which identified rapid expression of immediate early genes after SCI, followed by genes associated with inflammation, oxidative stress, DNA damage and cell cycle (2-6). Transcription factors, particularly those involved in cell damage and death, including nuclear factor kappa B, c-JUN and suppressor of cytokine signaling 3 were also observed to be upregulated (7). Several of the above findings have been proven by using experimental methods (8-10); thus, data from DNA microarray analysis can be reliable and useful for discovering novel targets for neuro-protective or restorative therapeutic approaches.

In addition, microRNAs (miRNAs) that can post-tran-scriptionally regulate the entire set of genes exhibited altered expression following traumatic SCI (11). Previous studies have suggested that miRNAs may act as mediators of neural plasticity (12) and possibly be involvement in neurodegeneration (13).

In the present study, microarray data (GSE45006) were used to screen differentially expressed genes (DEGs). Based on the screened DEGs, protein-protein interaction (PPI) network was then constructed and the roles of transcription factors and miRNAs in the regulation of DEGs were further investigated with the objective to expand the current knowlege on the molecular mechanisms of SCI.

## Materials and methods

### Microarray data

The raw microarray data (GSE45006) were downloaded from the Gene Expression Omnibus database (GEO; http://www.ncbi.nlm.nih.gov/geo/). The platform was GPL1355 [Rat230_2] Affymetrix Rat Genome 230 2.0 Array. Data from a total of 24 tissue samples from the epicenter area of normal (n=4) and injured (n=20) rat thoracic spinal cords (T7) were used, and the latter contained four samples from rats with spinal cord injury after one day, three days as well as 1, 2 and 8 weeks, respectively.

### Microarray data pre-processing and screening of DEGs

First, the extracted expression microarray data were standardized using the Robust Multiarray Averaging (RMA) method (14). Using the Bayesian model-based method provided by the Linear Models for Microarray (LIMMA) data package of R/Bioconductor (15), gene expression values in the experimental groups at the five time-points after spinal cord injury were compared with those in the normal samples. Genes with |log2 fold change|>1 and P<0.05 were regarded as DEGs. Subsequently, with reference to Zhang *et al* (16), DEGs that were significantly differentially expressed by at least two-fold were selected as the spinal cord injury tag genes (referred to as up-regulated genes and down-regulated genes below). The screened DEGs were submitted to the Database for Annotation, Visualization and Integrated Discovery (DAVID) for Kyoto Encyclopedia of Genes and Genomes (KEGG) pathway enrichment analysis using the module functional chart (P<0.05) (17).

### Screening of SCI-induced time-associated genes and functional annotation

Changes in gene expression levels reflected by the microarray may be caused by either biological factors or the background noise (4). To exclude the influence of background as far as possible, the standard deviation of the expression value of each gene was calculated. Assuming that a larger standard deviation cannot be solely caused by abiotic factors such as background noise, genes were screened according to the value of standard deviation by retaining those with top 30% standard deviations. Through comparing several times, screening the top 10, 15, 20 and 30% DEGs, it was confirmed that this threshold was able to sufficiently balance the specificity and sensitivity.

The Pearson correlation coefficient between the expression levels of screened gense and the time after spinal cord injury was calculated using R/Bioconductor software, with P=0.01 defined as the significant correlation level. As the sample size was 24 in the present study, the correlation coefficient was approximated to be >0.5 or <−0.5 at this significance level. Positively and negatively DEGs meeting this criterion were submitted to DAVID to analyze the enriched gene ontology (GO) biological processes.

### Construction of a protein-protein interaction (PPI) network

To elucidate the interaction of the DEGs, the Search Tool for the Retrieval of Interacting Genes/Proteins (STRING) database was utilized to build an interaction network of encoding products of DEGs (18). A STRING score of 0.4 was set as the reliability threshold. The obtained results were drawn into a network by Cytoscape software, version 2.8 (Institute of Systems Biology, Seattle, WA, USA). The degree of interaction of each gene in the network was calculated.

### Prediction of regulatory factors of DEGs

The DEGs were submitted to GeneCodis (19) to evaluate which transcription factors have binding sites to DEGs (data source, Transfac) at the significance level using the Fisher’s exact test, in order to predict whether the corresponding transcription factor is in an activated or suppressed state, taking the value of 0.05 divided by the number of tested transcription factors as the significance threshold. Similarly, Fisher’s exact test was used to evaluate which miRNAs enrich down-regulated DEGs to speculate which function they have in SCI, taking the value of 0.05 divided by the number of tested miRNAs as the significance threshold.

## Results

### Screening and biological pathway enrichment analysis of DEGs

In total, 806 upregulated DEGs and 549 downregulated DEGs were screened. According to the KEGG biological pathway enrichment analysis, it was found that the upregulated DEGs were significantly enriched in 13 pathways (P<0.05), including lysosome, complement and coagulation cascades and extracellular matrix-receptor interaction (Table I). However, none of the downregulated DEGs were enriched in any pathways.

### Gene expression over time after SCI

Correlation analysis revealed that the levels of 314 DEGs were enhanced with increasing time after SCI (correlation coefficient >0.5), while the expression levels of 253 DEGs were decreased over time (correlation coefficient <−0.5).

**Figure 1:**
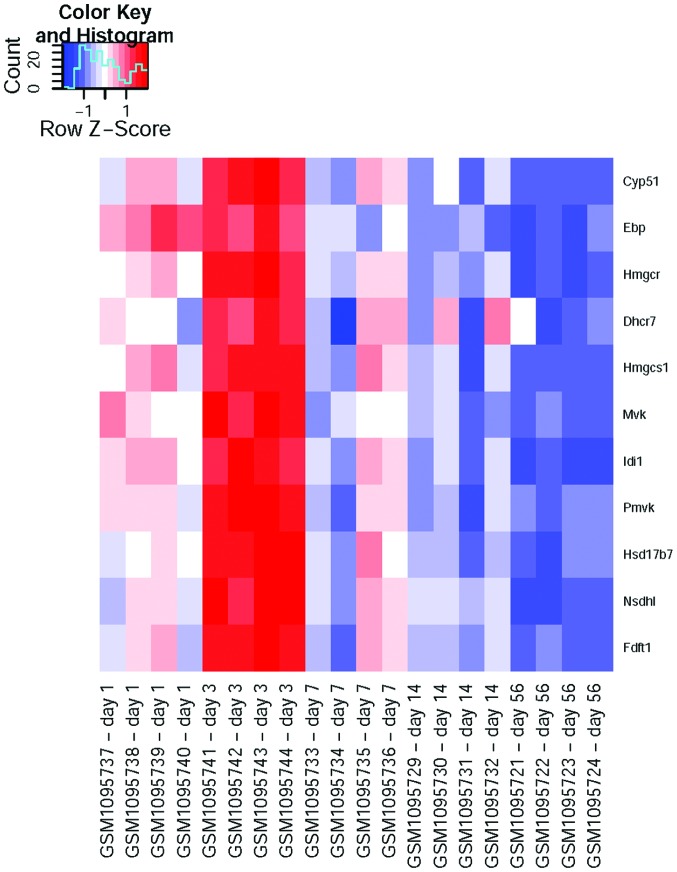
Expression profiles of injury-associated genes. The horizontal axis represents the time after spinal injury, and the vertical axis represents a specific gene (a darker red indicates a stronger upregulation in expression and a darker blue indicates a stronger downregulation in expression).

**Figure 2:**
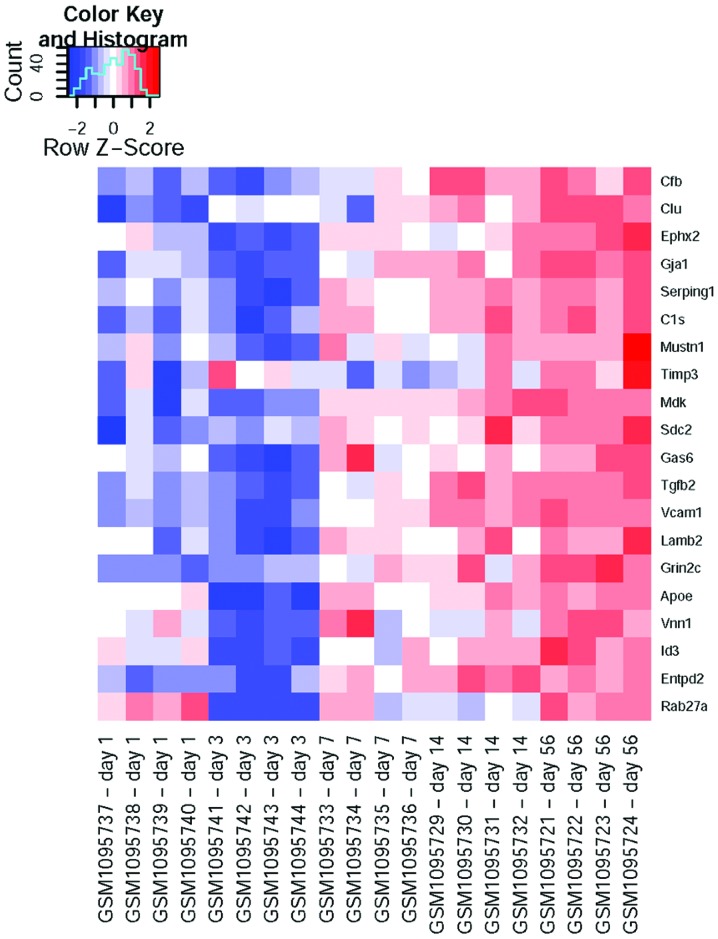
Expression profiles of cholesterol metabolism-associated genes. The horizontal axis represents time following spinal injury, and the vertical axis represents a specific gene (a darker red indicates a stronger upregulation in expression and a darker blue indicates a stronger downregulation in expression).

Through GO annotation, it was found that DEGs with expression levels negatively correlated with time after SCI were mainly cholesterol metabolism-associated genes (Table IIA), including *CYP51*, *EBP*, *HMGCR*, *DHCR7*, *HMGCS1*, *MVK*, *IDI1* and *FDFT1*, whereas those with expression levels positively correlating with time were mainly involved in injury response (Table IIB), including *SERPING1*, *C1S*, *ENTPD2* and *RAB27A*. According to the heatmap (Fig. 1), it was found that the expression levels of cholesterol metabolism-associated DEGs peaked on day three after injury and then dropped constantly, while the injury response-associated DEGs were gradually upregulated after injury and peaked at the 8th week (Fig. 2).

### Construction of a PPI network

**Figure 3:**
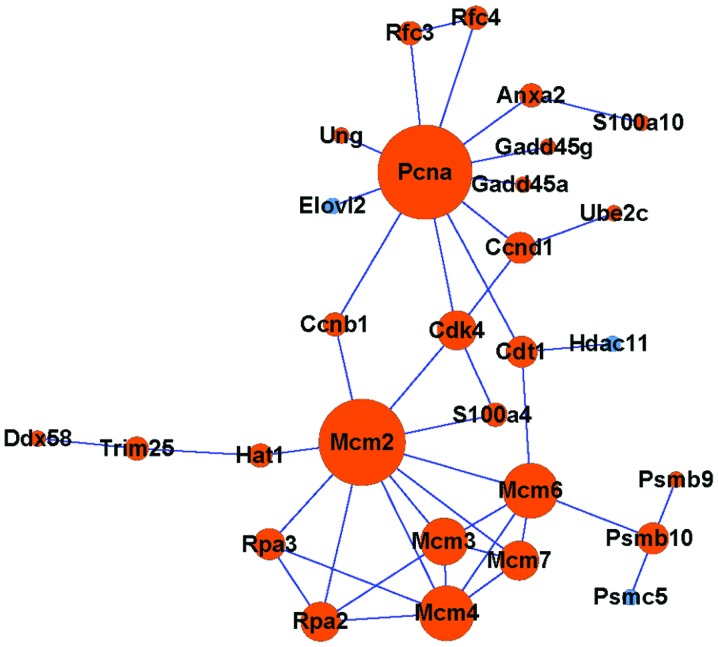
Sub-network of protein-protein interaction. The red circles represent upregulated proteins and the blue circles represent downregulated proteins. The size of a protein is determined by the degree of its connection to other proteins.

**Figure 4:**
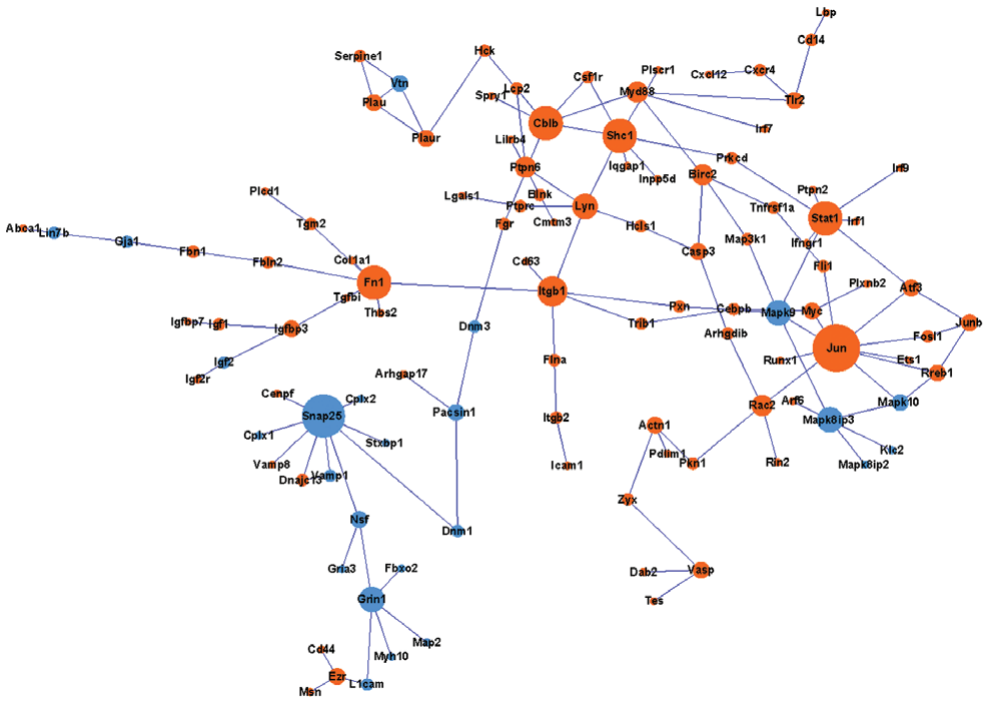
Sub-network of protein-protein interaction. The red circles represent upregulated proteins and the blue circles represent downregulated proteins. The size of a protein is determined by the degree of its connection to other proteins.

According to the constructed PPI network, there were at least two sub-networks, and most proteins in the two sub-networks were upregulated. *JUN* and *SNAP25* as well as *PCNA* and *MCM2* were the hubs of the two sub-networks, respectively (Figs. 3 and 4). Among them, *SNAP25* was downregulated, while *JUN*, *PCNA* and *MCM* were upregulated.

### Regulation of DEGs screened in rats with SCI

Transcription factors were observed to participate in the up- and downregulation of DEGs. A total of 185 and 1,215 transcription factors were screened for the up- and downregulated DEGs, respectively. Among them, the top three transcription factors with affinity for binding sites in the upregulated DEGs were SP1 (102 target DEGs, P=5.22477×10^−15^), MAZ (87 target DEGs, P=1.36×10^−141^) and LEF1 (81 target DEGs, P=5.58×10^−97^), respectively. The top three transcription factors with affinity for binding sites in the downregulated DEGs were LEF1 (87 target DEGs, P=6.05×10^−22^), E12 (85 target DEGs, P=1.14×10^−23^), and MAZ (79 target DEGs, P=2.89×10^−21^), respectively. LEF1 and SP1 were observed to have target DEGs that were involved in cholesterol-associated metabolism (e.g. *FDFT1* and *HMGCS1*) and in injury responses (e.g. *C1S* and *RAB27A*). Further transcription factors, NFAT, AP4, SREBP1 and STAT5B, were also observed to target upregulated DEGs that were involved in injury responses, and NFY, TATA and MEIS1 were observed to target downregulated DEGs that were involved in cholesterol metabolism.

In addition, 151 miRNAs were predicted for the down-regulated DEGs. miR-429 was indicated to regulate 26 downregulated DEGs (P=1.52×10^−10^), and miR-200a and miR-141 regulated 23 downregulated DEGs each, with P-values of 8.7×10^−8^ and 1.4×10^−8^, respectively. In addition, a number of miRNAs, including miR-16, miR-210, miR-15b, miR300-3p, miR-540, miR-325-5p and miR-487b, were observed to have target DEGs involved in cholesterol-associated metabolism, e.g. *IDI1* and *FDFT1*.

## Discussion

In the present study, *JUN*, *SNAP25*, *PCNA* and *MCM2* were the hub nodes in the constructed PPI network. The JUN family protein members c-JUN, JUNB and JUND are necessary for the assembly of the AP-1 (20) transcription factor complex. The major component, c-JUN, is highly induced in response to neuronal injury, which is mediated by C-JUN N-terminal kinase 1 (JNK) via phosphorylation (21,22). This explains for the upregulation of JUN observed in the present study, confirming the neuronal injury after SCI. *SNAP25* is a component of the trans-SNARE complex, relating to membrane fusion (23), which has been reported to ameliorate the sensory deficit in rats with SCI (24). The downregulation of *SNAP25* expression in the present study may therefore be associated with the sensory deficit after SCI.

*PCNA* is a DNA clamp that acts as a processivity factor for DNA polymerase delta with the help of *RFC* in eukaryotic cells; thus, it is essential for DNA replication and repair (25-27). *PCNA* was observed to be upregulated in the present study, which is consistent with the results of previous studies by Ding *et al* (28) and Di Giovanni *et al* (6) who have reported an upregulation in *PCNA* expression after SCI by using western-blot and RT-qPCR analyses. Mini-chromosome maintenance protein 2 (MCM2) protein is one of the highly conserved MCMs, which form the hexameric protein complex that is involved in the initiation and the elongation of eukaryotic genome replication, particularly the formation and elongation of the replication fork (29,30). The upregulation of *PCNA* and *MCM2*, two DNA replication-associated factors, indicates the effort of cells to repair DNA and regenerate themselves, further demonstrating neuronal damage and death after SCI. Di Giovanni *et al* (6) have proven that *PCNA*, together with other cell cycle-associated genes, is involved in the neuronal damage and subsequent cell death after SCI.

Several studies have reported the disturbed cholesterol metabolism in spinal cord-injured patients (31,32). In the present study, the downregulation of cholesterol metabolism-associated genes over time was observed following SCI. Previous studies have reported the regulatory role of miRNAs in lipid and cholesterol metabolism, particularly miR-33 (33,34). According to the present study, several miRNAs were observed to target cholesterol metabolism-associated DEGs, including miR210, miR300-3p, miR-325-5p, miR-487b and miR-16. A common target DEG of the former four was *IDI1*, and that of the latter was *FDFT1*, which are cholesterol biosynthetic enzyme genes that have also been reported to be expressed at reduced levels in the stroke-prone hypertensive rat (SHRSP) with lower total cholesterol levels in the serum. Therefore, these miRNAs are also indicated to have important roles in the regulation of cholesterol and sterol biosynthesis after SCI, which requires further experimental verification. miR-429, miR-141 and miR-200a belong to the same miR-200 family. Benoit *et al* (35) have reported the upregulation of rno-miR-200a in rats on a high-fat diet. Thus, it is presumed that there may be a certain correlation between rno-miR-200a and the downregulation of cholesterol metabolism-associated genes over time. However, no targets of miR-200a, miR-429 and miR-141 were observed in the cholesterol metabolism-associated DEGs observed in the present study, which may be attributed to the small sample size of the microarray used. Hence, whether this miRNA family may have a regulatory role in lipid metabolism, particularly in the cholesterol/sterol metabolism, requires further investigation.

The transcription factors LEF1 and SP1 were observed to be associated with the regulation of the DEGs that were involved in cholesterol-associated metabolism and in injury responses; thus, it may be presumed that these two transcription factors have critical regulatory roles in gene expression after SCI. SP1 is a ubiquitous transcription factor. It has been reported to activate the LCAT promoter, which modulates the transportation rate of cholesteryl ester to the liver (36). Furthermore, it was observed that one of the target DEGs of SP1 was *RAB27A*, which is involved in the injury response, suggesting its role in the regulation of injury-associated DEGs after SCI. This agrees with the finding that SP1 or SP1-associated proteins are involved in regulating the expression of peripherin intermediate filament gene, which is activated after nerve injury via binding to the intron 1 site (37). Thus, whether SP1 functions in the same way in regulating injury-associated genes after SCI should be further validated. LEF1 is a member of the LEF-1/TCF family of transcription factors, which functions by interacting with cytosolic β-catenin to form a transcription complex that activates the Wnt signaling pathway (38). Functional TCF/LEF1 signaling has been reported to regulate lipid metabolism (39). In the present study, LEF1 was observed to downregulate DGEs that were involved in cholesterol-associated metabolism; thus, it is consistent with the previous finding that the Wnt signaling pathway is attenuated after SCI (40). In addition, LEF1, which participates in the Wnt signaling pathway, is highly expressed in the oligodendrocyte precursor cells (OPCs) after neonatal brain injury (41). In the present study, one of the target DEGs of LEF1, *C1S*, which is involved in complement systems, was observed to be upregulated, confirming its role in injury responses after SCI.

In conclusion, the present study revealed that expression of cholesterol metabolism-associated DEGs was downregulated over time, while injury-associated DEGs were upregulated over time after SCI. Furthermore, the hub genes *PCNA*, *MCM2*, *JUN* and *SNAP25* presumably have critical roles in rats with SCI, and the transcription factors LEF1 and SP1 may be important for the regulation of cholesterol metabolism and injury responses after SCI.

## Figures and Tables

**Table I:** Enriched pathways of upregulated differentially expressed genes.

Pathway	P-value	Genes	Benjamini
rno04142: Lysosome	1.31×10^−8^	ARSB, GM2A, LGMN, HEXA, HEXB, ACP5, CTSA, CTSL1, CD68, LAPTM5, SCARB2, MAN2B1, TCIRG1, CTSZ, LIPA, PLA2G15, GUSB, CD63, MANBA, CTSK, IGF2R, CTSD, CTSC, CTSB, CLN5	1.93×10^−6^
rno04610: Complement and coagulation cascades	1.40×10^−7^	C3AR1, C5AR1, F13A1, C1R, SERPING1, C1S, C1QC, PLAUR, C1QA, C1QB, THBD, SERPINE1, CFH, C2, CFD, PROS1, PLAU, CR1L	1.04×10^−5^
rno04512: ECM-receptor interaction	1.31×10^−6^	COL4A1, COL3A1, ITGB1, COL5A2, COL5A1, CD47, SDC1, CD36, CD44, COL6A3, COL6A2, COL6A1, COL1A1, LAMC1, THBS2, SPP1, THBS4, FN1	6.48×10^−5^
rno04062: Chemokine signaling pathway	1.78×10^−5^	CXCL1, ADCY4, CCL3, CCL2, FGR, CCL9, NFKBIA, PF4, CXCL12, CCL7, PXN, CXCL10, RAC2, CXCR4, RHOC, SHC1, LYN, HCK, STAT1, VAV1, PRKCD, GNGT2, CXCL14, CXCL16, CX3CR1	6.58×10^−4^
rno04510: Focal adhesion	2.01×10^−5^	COL3A1, ITGB1, PXN, RAC2, COL6A3, COL6A2, COL6A1, RHOC, SHC1, ZYX, THBS2, SPP1, FN1, THBS4, COL4A1, IGF1, ACTN1, BIRC2, COL5A2, VAV1, VASP, COL5A1, FLNA, CCND1, JUN, COL1A1, LAMC1	5.93×10^−4^
rno03030: DNA replication	8.23×10^−5^	RPA2, RFC3, MCM7, RFC4, PCNA, MCM2, MCM3, MCM4, RPA3, MCM6	2.03×10^−3^
rno04650: Natural killer cell mediated cytotoxicity	3.31×10^−4^	ICAM1, PTPN6, ITGB2, VAV1, HCST, CD48, CASP3, RAC2, FCGR2B, FCER1G, SHC1, FCGR3A, IFNGR2, IFNGR1, TYROBP, LCP2	6.97×10^−3^
rno04670: Leukocyte transendothelial migration	5.04×10^−4^	ICAM1, NCF4, ACTN1, ITGB2, MMP2, ITGB1, VAV1, CXCL12, VASP, PXN, CYBA, CYBB, EZR, RAC2, CXCR4, RHOC, MSN	9.29×10^−3^
rno04666: Fc gamma R-mediated phagocytosis	9.50×10^−4^	PTPRC, LYN, HCK, ARF6, ARPC5, VAV1, PRKCD, VASP, ARPC1B, RAC2, FCGR2B, ARPC3, FCGR1A, INPP5D	1.55×10^−2^
rno04060: Cytokine-cytokine receptor interaction	1.11×10^−3^	CCL3, CCL2, LTBR, TNFRSF12A, IL18, TGFBR2, PF4, TNFSF13, TNFSF12, CXCL12, IL17RA, CXCL10, TNFRSF1A, CXCL14, CXCR4, IL10RB, LOC688637, CXCL16, CX3CR1, IFNGR2, CSF2RA, IFNGR1, CSF1R	1.63×10^−2^
rno04623: Cytosolic DNA-sensing pathway	3.22×10^−3^	DDX58, IRF7, IL18, RIPK3, PYCARD, NFKBIA, IL33, CASP1, CXCL10	4.25×10^−2^
rno05322: Systemic lupus erythematosus	3.59×10^−3^	ACTN1, C1R, C1S, C1QC, RT1-DA, RT1-BB, C1QA, C1QB, FCGR2B, FCGR1A, SNRPB, C2, FCGR3A	4.33×10^−2^
rno04110: Cell cycle	3.66×10^−3^	TGFB3, MCM2, MCM3, CDK4, MCM4, TGFB1, MCM6, CCNB1, CCND1, MCM7, GADD45G, PCNA, MAD2L2, MGC112830, MYC, GADD45A	4.09×10^−2^

The Benjamini value is a parameter generated during adjustment of the P-value for multiple comparisons.

**Table II:** Enriched GO biological processed of DEGs.

A, Enriched GO biological processes of downregulated DEGs			
GO term and function	P-value	DEGs	Benjamini
0006695: Cholesterol biosynthetic process	2.48×10^−13^	CYP51, EBP, HMGCR, DHCR7, HMGCS1, MVK, IDI1, PMVK, HSD17B7, NSDHL, FDFT1	3.33×10^−10^
0016126: Sterol biosynthetic process	2.16×10^−12^	CYP51, EBP, HMGCR, DHCR7, HMGCS1, MVK, IDI1, PMVK, HSD17B7, NSDHL, FDFT1	1.45×10^−9^
0008203: Cholesterol metabolic process	5.61×10^−12^	CYP51, EBP, HMGCR, HMGCS1, PMVK, FDFT1, SREBF2, SQLE, DHCR7, MVK, IDI1, HSD17B7, NSDHL, VLDLR	2.52×10^−9^
0016125: Sterol metabolic process	1.63×10^−11^	CYP51, EBP, HMGCR, HMGCS1, PMVK, FDFT1, SREBF2, SQLE, DHCR7, MVK, IDI1, HSD17B7, NSDHL, VLDLR	5.50×10^−9^
0008610: Lipid biosynthetic process	6.70×10^−11^	SCD1, CYP51, EBP, HMGCR, FA2H, NDUFAB1, HMGCS1, ACLY, ACSS2, PMVK, FDFT1, SREBF2, FAR1, AGPS, DHCR7, RGD1560015, MVK, PCYT2, AGPAT4, IDI1, HSD17B7, NSDHL	1.80×10^−8^
0006694: Steroid biosynthetic process	2.24×10^−9^	CYP51, EBP, HMGCR, RGD1560015, DHCR7, HMGCS1, MVK, IDI1, PMVK, HSD17B7, NSDHL, FDFT1	5.03×10^−7^
0008202: Steroid metabolic process	3.08×10^−8^ 5.91×10^−6^	CYP51, EBP, HMGCR, HMGCS1, PMVK, FDFT1, SREBF2, SQLE, DHCR7, RGD1560015, MVK, IDI1, HSD17B7, NSDHL, VLDLR	

B, Enriched GO biological processes of upregulated DEGs			
GO term and function	P-value	DEGs	Benjamini
0010033: Response to organic substance	1.24×10^−7^	RT1-M3-1, GJA1, CDH1, C1S, TIMP2, MDK, CXCL12, TIMP3, SDC2, TGFB2, B2M, GSTM2, SORBS1, APOE, SULT1A1, LCAT, GPX3, CD4, BOC, NR1H3, SREBF1, TXNIP, PLAT, CFB, SLC22A8, IGF2, NR4A3, STAT1, PRKCD, CYP7B1, CDKN1B, ID2, PTGDS, BTG1, ALDH2, NFE2L2, ID3, IGFBP2, MGST1	2.38×10^−4^
0042493: Response to drug	2.84×10^−5^	TXNIP, SREBF1, ATP1A1, CDH1, IGF2, TIMP2, STAT1, MDK, PRKCD, MMP12, TGFB2, B2M, CYP7B1, CDKN1B, GPX3, SLC22A5, IGFBP2, MGST1	2.68×10^−2^
0009611: Response to wounding	7.28×10^−5^	CFB, CLU, EPHX2, GJA1, SERPING1, C1S, MUSTN1, TIMP3, MDK, SDC2, GAS6, TGFB2, VCAM1, LAMB2, GRIN2C, APOE, VNN1, ID3, ENTPD2, RAB27A	4.54×10^−2^
0042127: Regulation of cell proliferation	9.35×10^−5^	IFITM3, CLU, PAX6, GJA1, SOX4, PRRX2, IL34, TIMP2, DDR2, CXCL12, TGFB2, VCAM1, EDNRB, APOE, HEY2, TXNIP, CRIP2, STAT1, MMP12, CYP7B1, CDKN1B, ID2, DBP, BTG1, ID3, PMP22	4.38×10^−2^
0009725: Response to hormone stimulus	1.01×10^−4^	PLAT, TXNIP, SREBF1, GJA1, IGF2, NR4A3, STAT1, PRKCD, MDK, TIMP3, CXCL12, TGFB2, CDKN1B, PTGDS, SORBS1, BTG1, SULT1A1, LCAT, GPX3, ALDH2, IGFBP2, NR1H3	3.79×10^−2^
0009719: Response to endogenous stimulus	1.88×10^−4^	PLAT, SREBF1, TXNIP, GJA1, IGF2, NR4A3, STAT1, PRKCD, MDK, TIMP3, CXCL12, TGFB2, CDKN1B, PTGDS, SORBS1, BTG1, SULT1A1, LCAT, GPX3, ALDH2, IGFBP2, MGST1, NR1H3	5.82×10^−2^

The Benjamini value is a parameter generated during adjustment of the P-value for multiple comparisons. DEG, differentially expressed gene; GO, gene ontology.
